# Intrahepatic cholestasis of pregnancy: observational study of the treatment with low-dose ursodeoxycholic acid

**DOI:** 10.1186/s12876-015-0324-0

**Published:** 2015-07-29

**Authors:** Titta Joutsiniemi, Susanna Timonen, Maria Linden, Pia Suvitie, Ulla Ekblad

**Affiliations:** Department of Obstetrics and Gynaecology, Turku University Central Hospital, Kiinamyllynkatu 4-8, 20520 Turku, Finland

**Keywords:** Pregnancy, Intrahepatic cholestasis, Ursodeoxycholic acid

## Abstract

**Background:**

To exam the biochemical, obstetric management and pregnancy outcome in women with intrahepatic cholestasis of pregnancy (ICP) and treatment with ursodeoxycholic acid (UDCA).

**Methods:**

Pregnancy outcome in patients with ICP (*N* = 307) was studied and patients treated with UDCA (*N* = 208) vs. no UDCA were compared. The data of the antenatal visits, deliveries and neonatal outcome of 307 pregnancies with ICP was collected from the hospital computerized delivery room log book. UDCA was used in 208 pregnancies. The diagnosis was made by maternal pruritus and elevation of total fasting bile acid (BA) (>6 μmol/l) and elevation of serum alanine aminotransferases (ALT) (>45 U/l). Maternal and neonatal data was analysed and data of the patients who used UDCA during pregnancy was analysed separately and compared with the data from patients without medication.

**Results:**

UDCA was well tolerated. Mothers receiving UDCA had ICP diagnosed five weeks earlier than mothers without medication. At the diagnosis, levels of total BA and ALT were higher in the group using UDCA compared to the group without medication. Most deliveries were induced and perinatal outcome was good. Apgar scores at 5 min were significantly lower in UDCA group (*p* < 0.05), but fetal umbilical artery pH values were similar in both groups (*p* > 0.05). There were 30 patients with total BA > 40 μmol/l at diagnosis, 24 with UDCA and 6 without medication and those deliveries were induced soon after diagnosis. The preterm labour was also more common in these patents (*p* < 0.05). Women with preterm babies had significantly early onset pruritus and ICP was diagnosed earlier. Serum ALT and total BA levels were significantly higher in those pregnancies at diagnosis and also at first control.

**Conclusions:**

Preterm labour was associated in severe ICP (total BA > 40 μmol/l), ALT levels were also significantly higher and ICP was diagnosed earlier (*p* < 0.05). Apgar scores were lower in preterm babies (*p* < 0.05), but umbilical artery pHvalues were not significantly lower. UDCA was well tolerated by pregnant women. With low-dose UDCA treatment the obstetric outcome was good. We still recommend careful obstetrical follow-up.

## Background

Intrahepatic cholestasis of pregnancy (ICP) is a condition of severe itching combined with elevated liver function tests (elevated fasting serum bile acids or/and elevated serum transaminases) in previously healthy pregnant women. It occurs typically during the second or third trimester of pregnancy and symptoms relief spontaneously within 2 to 3 weeks after delivery. In Europe, it affects approximately 10 to 150 per 10000 pregnancies [1]. The etiology of ICP is still unknown but its pathogenesis appears to be related to increased sex hormone synthesis and their altered liver metabolism during pregnancy [2]. Also environmental factors and genetic predisposition may be involved [2]. Variations in risk alleles for ICP in the two genes primarily responsible for bile formation have been found. *The phosphatidyl choline floppase ABCB4 (MDR3)* and the bile salt export pump *ABCB11* have key roles for common variations [3].

ICP resolves after delivery but it has been associated with high incidence of fetal complications. It increases the risk of preterm delivery, meconium excretion, respiratory distress syndrome and sudden intrauterine death [4]. Glantz et al. (2004) reported a 1–2 % increase in the risk of spontaneous preterm labour, asphyxial events or meconium staining of the amniotic fluid and/or placenta and membranes for every additional μmol/L of maternal serum bile acids [5]. In the same study no increase in adverse outcomes was reported if the maternal serum fasting bile acids were below 40 μmol/L. The risk of spontaneous preterm labour is increased in ICP pregnancies (19–60 %) [6]. Nowadays the majority of preterm deliveries are iatrogenic reflecting the practice of elective induction of labour in ICP cases with the aim of reducing the risk of fetal complications. Patients with ICP are considered as high risk patients and the timing of delivery should be decided outweighing the risk of prematurity and ICP complications. Routinely induction of labour is recommended for women with ICP after 37 pregnancy week [7]. The cesarean section rate is not increased by the early induction of labor [8, 9].

The treatment of ICP has mainly been symptomatic because the pathophysiology is still unresolved. Phenobarbital, cholestyramine, S-Adenosyl-L-methionine, dexamethasone and ursodeoxycholic acid have been used [10–13]. It has been shown that UDCA causes a significant reduction in liver function tests in ICP [13, 14]. Gurung et al. (2013) concluded in the Cochrane collaboration that UDCA significantly improves pruritus and fewer instances of fetal distress/asphyxial events were seen in the UDCA groups when compared with placebo but the difference was not statistically significant [15]. Bacq et al. [16] found UDCA have a several benefits for mothers, fetuses as well as for newborns.

We examined the pregnancy outcome and maternal liver function tests after using UDCA on Finnish patients. ICP patients were treated in clinical setting with UDCA and an observational retrospective study in ICP and UDCA was done.

## Methods

The study was performed in Turku University Central Hospital between 2000 and 2005. The diagnostics criteria and the treatment protocol are equal nowadays. During the study period there were 307 women with ICP. UDCA was used in 208 of these pregnancies. Thirty-four women had two pregnancies during this period, so the number of patients was 273. In this study there were 14 twin pregnancies, all together 321 newborns. In the UDCA group there were 13 twin pregnancies, all together 221 newborns. Ninety nine mothers received no medication. In the group without medication there was one twin pregnancy and all together 100 newborns.

The diagnosis was made by maternal itching in pregnancy with other causes of cholestasis excluded and elevation of total fasting bile acid (>6 μmol/l) and elevation of serum alanine aminotransferases (>45 U/l).

Patients started UDCA medication according to our standard protocol usually 450 mg/day. All participating patients fulfilled the diagnostic criteria, but those who only had mild symptoms or only slightly elevated liver function tests did not started UDCA. Also those whose laboratory values were high and labour induction was started within a few hours did not have the medication.

A study questionnaire data on maternal age, gravidity, parity, complications of earlier pregnancies, body mass index, and use of drugs, medical history, smoking and heredity of ICP was collected. All follow-up visits in the antenatal clinic and the dose and side-effects of UDCA were also recorded. No formal scale was used to show improvement of pruritus. All patients whose laboratory values were improved also reported decreased pruritus. Patients whose laboratory values or symptoms got worse, labour were induced or dose of UDCA was increased. Eighteen patients used UDCA 600 mg/day and one patient 900 mg/day. The data of follow-up visits included gestational age, blood pressure, urine samples and the control of biochemical tests including ALT and total BA. The fetal ultrasound findings and cardiotocography (ctg) were also registered.

Obstetric outcome was evaluated. Gestational age at delivery, induction of labour, duration of labour and obstetrical managements was recorded. Neonatal outcome (Apgar scores (1, 5 and 15 min), birth weight, pH and base excess values from umbilical artery blood) was collected. Maternal and neonatal data was analysed and data of the patients who used UDCA during pregnancy was analysed separately. Also pregnancies with total bile acids > 40 μmol/l at diagnosis or preterm birth were analysed separately.

The statistical analysis was made with T-test, Mann–Whitney test and Chi-Square test. P-values less than 0.05 were considered statistically significant.

The study protocol was approved by the Ethical Committee of the University and the Hospital. The informed consent was not obtained, because the study was retrospective.

## Results

Patients’ characteristics are gathered in the Table 1. Age, parity, smoking, body mass index and ICP heredity were similar between these two groups. The mean gestational age at the beginning of pruritus was 33.6 weeks in the whole group, 32.2 in the UDCA group and 36.8 in the group without medication (*p* < 0.05). Mothers receiving UDCA during pregnancy had ICP diagnosed 5 weeks earlier than mothers without medication (*p* < 0.05). In most cases without UDCA, the onset of ICP was in the last weeks of pregnancy and these mothers did not require treatment, either because the symptoms were mild or because the pregnancy was terminated. The abdominal ultrasound was made to patients suffering from high levels of liver enzymes especially increased ALT to exclude other liver pathology. Gall stones were detected by ultrasonograhpy in 4 % (*N* = 8) of the mothers using UDCA and in 1 % (*n* = 1) in the group without medication for ICP. None of the patients were operated due to cholelithiasis during pregnancy.

**Table 1:** Patients’ characteristics comparing ursodeoxycholic acid treatment and no medical treatment in ICP. Values are given as numbers (%) or mean [SD]

	ALL *n* = 307	No medication *n* = 99	UDCA *n* = 208	P
Maternal age (years)	29.6 [5.2]	28.8 [4.6]	29.9 [5.4]	NS
Smoking	26 (8 %)	12 (12 %)	14 (7 %)	NS
Body mass index	23.5 [4.1]	22.9 [3.7]	23.8 [4.2]	NS
Former deliveries (range)	1.0 (0–8)	1.0 (0–8)	0.8 (0–5)	NS
ICP in earlier pregnancy	92 (30 %)	27 (27 %)	65 (31 %)	NS
Heredity	7 (2 %)	3 (3 %)	4 (2 %)	NS
Beginning of itching (wks)	33.6 [3.9]	36.8 [2.5]	32.2 [3.6]	*P* < 0.05
ICP diagnosed (wks)	34.8 [3.5]	38.0 [1.5]	33.3 [3.1]	*P* < 0.05
Bile acid at diagnosis (μmol/l)	19.2 [24.9]	16.7 [26.7]	20.5 [24.0]	NS
ALT at diagnosis (U/l)	152.8 [166.1]	96.4 [106.6]	179.2 [181.9]	*P* < 0.05
Abdominal ultrasound	35 (11 %)	4 (4 %)	31 (15 %)	*p* < 0.05
Bile stones	9 (3 %)	1 (1 %)	8 (4 %)	NS

The medication with UDCA was started for 208 patients and the mean dose of UDCA was 450 mg/day (range 150–900). The mean gestational age at the diagnosis was 33.3 weeks and at the first control visit 34.8 weeks. Most patients started UCDA medication at the first visit in the maternal care unit (*N* = 196) and their first control was at 34.2 gestational week. Twelve patients started medication later in the pregnancy because ICP was mild at the diagnosis. The symptoms got worse and the liver enzymes were higher later in the pregnancy and the medication was started. Only 1.4 % of the patients had side effects from the medication. Two patients had abdominal pain and one reported nausea.

**Fig. 1:**
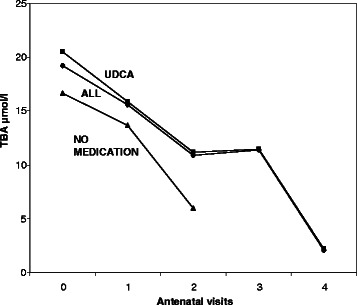
Total bile acid (TBA) concentration (μmol/l) with patients with UDCA (■), all patients (♦) and patients without medication (▲) at the diagnosis (0) and at visits (1–4) in the antenatal clinic. The statistical analysis was calculated during two weeks. All patients: Decrease in TBA levels was statistically significant between at diagnosis and second antenatal visit and also between first and second antenatal visit (*p* < 0.05). UDCA group: Decrease in TBA levels was statistically significant between at diagnosis and first antenatal visit and between first and second antenatal visit (*p* < 0.05)

**Fig. 2:**
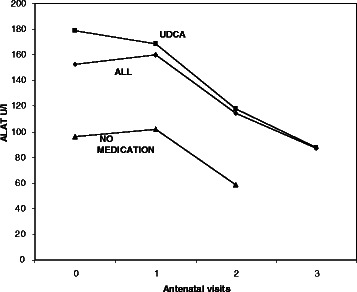
Concentration of alanine aminotransferase (U/l) with patients with UDCA (■), all patients (♦) and patients without medication (▲) at the diagnosis (0) and at visits (1–4) in the antenatal clinic. The statistical analysis was calculated during two weeks. All patients and UDCA group: Decrease in ALT levels was statistically significant between at diagnosis and second antenatal visit and also between first and second antenatal visit (*p* < 0.05)

**Fig. 3:**
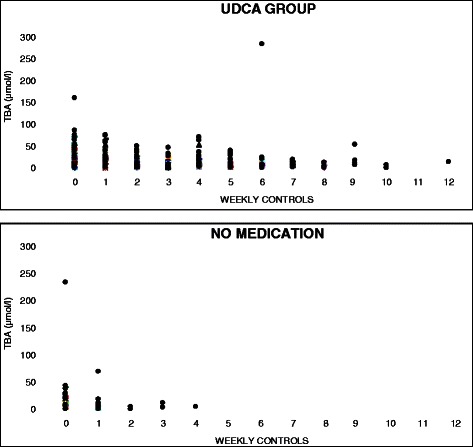
Total bile acid concentrations (μmol/l) in the group using UDCA and in the group without the medication

**Fig 4:**
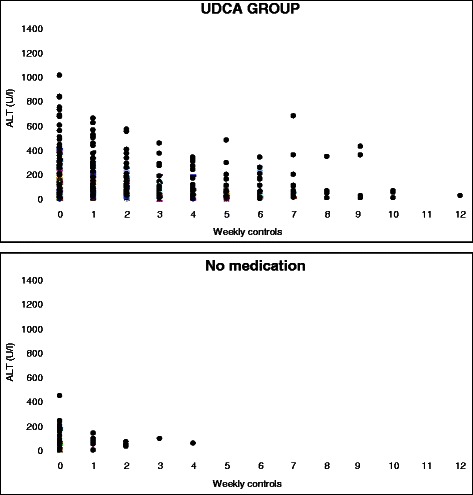
Plasma alanine aminotransferase concentrations (U/l) in the group using UDCA and in the group without the medication

At the diagnosis levels of total BA and serum ALT were higher in the group using UDCA compared to the group without the medication, but only the difference between the levels of ALT was statistically significant (*p* < 0.05). Figs. 1 and 2 represent these values at the diagnosis and at the visits in the antenatal clinic. Both total bile acid level and concentrations of ALT begun to decrease after starting the medication (Figs. 3 and 4). The decline of total BA values between diagnosis and the control visits was statistically significant in UDCA group (*P* < 0.05). Serum ALT values begin to decrease significantly after first control visit (*p* < 0.05). Also in the group without the medication a decrease in serum levels of BA acids and ALT was recorded probably because the patients with a more severe ICP delivered already during the first weeks of the study period and the number of women coming to the last visits was small and the ICP was mild. However that was not statistically significant.

Obstetric and neonatal characteristics are presented in Table 2. Mothers using UDCA delivered on the mean of 37 gestational week and mothers without medication on the mean of 38 gestational week (*p* < 0.05). There were more preterm deliveries (<37 weeks) in the group using UDCA due to severity of ICP (*p* < 0.05). Most of the deliveries were induced and labour induction rate was higher with UDCA users (*p* < 0.05). Caesarean section rate in our hospital during the study period varied between 13.9 and 17.4 % and in the study cohort the rate was 15 %. The vacuum extraction rate was 5.8–7.3 % in the hospital and in this study 5.5 %. The perinatal outcome was good and there were no perinatal deaths in our study material. Apgar scores at 5 min were significantly lower in UDCA group (*p* < 0.05), but fetal umbilical artery pH values were similar in both groups (*p* > 0.05). The rate of admissions to the neonatal unit was higher for children in the UDCA group (30 % vs 11 %) mostly due to preterm labour (Table 3) which was more common in UDCA users (*p* < 0.05). Women with preterm babies had significantly early onset pruritus and ICP was diagnosed earlier. Serum ALT and total BA were significantly higher in those pregnancies at diagnosis and also at first control. Also new-borns’ Apgar scores were significantly lower (*p* < 0.05), but umbilical artery pH values were equal.

**Table 2:** Obstetrics and neonatal outcome. Values are given as numbers (%) or mean [SD]

Characteristic	All (*n* = 307)	UDCA (*n* = 208)	No medication (*n* = 99)	P
Time of delivery (weeks)	37.9 [1.6]	37.4 [1.5]	38.9 [1.2]	*p* < 0.05
Preterm delivery (<37 weeks)	57 (19 %)	53 (25 %)	4 (4 %)	*p* < 0.05
Gemini	14 (5 %)	13 (6 %)	1 (1 %)	*p* > 0.05
Induction of the labour	222 (72 %)	143 (69 %)	79 (80 %)	*p* < 0.05
Normal vaginal delivery	245 (80 %)	161 (77 %)	84 (85 %)	NS
Children	(*n* = 321)	(*n* = 221)	(*n* = 100)	
Female	144 (45 %)	98 (44 %)	46 (46 %)	NS
Male	177 (55 %)	123 (56 %)	54 (54 %)	NS
Weight	3344 [531]	3262 [553]	3525 [428]	*p* < 0.005
Apgar score (5 min)	8.8 [1.0]	8.7 [1.0]	9.0 [0.9]	*p* < 0.05
Fetal Artery pH	7.28 [0.09]	7.28 [0.09]	7.28 [0.10]	NS
Fetal Artery pH < 7.05	2 (1 %)	2 (1 %)	-	NS
Fetal venous BE, mmol/l	−4.1 [3.2]	−4.0 [3.2]	−4.3 [3.2]	NS
Fetal BE < −12 mmol/l	4 (1 %)	3 (1 %)	1 (1 %)	NS

**Table 3:** Indications for the new-borns’ treatment of NICU

Indications	No medication *N* = 100	UDCA *N* = 122	All *N* = 321
Prematurity	2 (2 %)	25 (11 %)	27 (8 %)
Hypoglycaemia	3 (3 %)	16 (7 %)	19 (6 %)
Infection	4 (4 %)	8 (4 %)	12 (4 %)
Breathing prob.^a^	1 (1 %)	5 (2 %)	6 (2 %)
Asphyxia	-	3 (1 %)	3 (1 %)
Exposure to drugs	1 (1 %)	2 (1 %)	3 (1 %)
Weight gain prob	-	2 (1 %)	2 (1 %)
Other	-	6 (3 %)	6 (2 %)
	11 (11 %)	67 (30 %)	78 (24 %)

^a^including wet lung or RDS

Statistical analysis was not done because of small number of the patients

There were 30 patients with severe ICP (total BA > 40 μmol/l), 24 with UDCA and 6 without medication. Four patients got increased UDCA dose to prevent preterm delivery. Eight patients delivered spontaneously, 22 were induced. The preterm labour was also more common in these patients (*p* < 0.05). Almost half of these babies, fourteen, had a treatment period in NICU.

## Discussion

Intrahepatic cholestasis of pregnancy has been associated with higher frequency of fetal distress and even intrauterine death. The treatment of ICP has been mainly symptomatic as no specific treatment exists.

UDCA is hydrophilic bile acid and used for the treatment of various cholestatic disorders. Currently, UDCA is the most promising treatment for ICP. It is well-tolerated by mothers and no adverse effects in newborns have been observed. Palma et al. [17] repoted UDCA improved significantly serum biochemistry in patients with ICP. This study was followed by three small randomised controlled trials showing a significant reduction of pruritus and liver function tests after using UCDA in ICP [14, 18, 19]. Also in a Swedish study UDCA significantly reduced aminotransferase and bilirubin levels in all treated women and there was a significant reduction of pruritus and bile acids in women with serum bile acid levels exceeding 40 μmol/l at inclusion [5]. In recent meta-analysis UDCA was also effective in reducing pruritus and improving liver test results in patients with ICP [16]. According to Cochrane database there is insufficient evidence to indicate many treatment alone or in combination in treating ICP patients [15]. There is also a new meta-analysis which includes both non- randomized studies and randomized controlled trials [20]. According to that study UDCA treatment should be recommended for women with ICP and it reduces adverse maternal and fetal outcome [20]. In our study both total bile acid levels and concentrations of alanine aminotransferase begun to decrease after starting the medication. However patients’ total BA values decreased significantly after starting the medication and ALT levels decreased significantly after first control visit.

Mazzella et al. [21] reported no adverse reactions of high dose UDCA (1.5–2 g/d) and concluded that the high-dose UDCA treatment appeared to improve both biochemical and clinical parameters of cholestasis and seemed to be completely safe for the fetus. Zapata et al. [22] concluded that UDCA was well tolerated by pregnant women and no adverse effects were detected in 26 infants followed up for a mean of 6 years after delivery. UDCA was beneficial in ICP patients in terms of improving maternal pruritus, liver tests and also the final outcome of pregnancy. UDCA was also well tolerated and no adverse side effects were detected neither in mothers nor newborns followed up for 3 months after birth. An updated follow-up of 26 of these children with ages up to 12 years old confirmed a normal growth and development and a lack of disease events attributable to UDCA [22]. In earlier studies the dose of UDCA has varied: Diaferia et al. and Nicastri et al. used 600 mg/day, Palma et al. and Glantz et al. used a higher dose of UDCA 1000 mg/day and Mazzella et al. even 2 g/day [13, 14, 18, 19, 21].

In our study there were more preterm deliveries (<37 weeks) in the group using UDCA due to the earlier onset and severity of ICP. The rate of admissions to the neonatal unit was higher for children in the UDCA group due to preterm labour. Pregnancies complicated by ICP had a significantly increased risk for iatrogenic and spontaneous preterm delivery (Puljic et al. [23]). Nowadays the majority of ICP patients’ deliveries are induced preterm with the aim of reducing the risk of adverse fetal outcome. One cohort study suggests that induction of labour may reduce intrauterine fetal death compared with expectantly management [24]. Rioseco et al. concluded that labour induction in ICP patients was beneficial [6]. Roncaglia [9] et al. concluded that elective delivery at 37 weeks in addition to monitoring fetal well-being can significantly reduce the stillbirth rate without increasing caesarean section rate. In a recent study of Puljic et al. [23] demonstrated that delivery at 36 weeks’ gestation reduced the perinatal mortality risk as compared with expectant management. They concluded timing of delivery has to take into account both the reduction in stillbirth risk balanced with the morbidities associated with preterm delivery [23].

However, preterm babies have an increased risk of respiratory distress syndrome (RDS) and the risk is even higher in elective cesarean section than in induced vaginal delivery [25]. In addition, maternal cholestasis may predispose the newborn to unexpected respiratory distress syndrome [26]. Zecca et al. concluded that the incidence of RDS in newborns from cholestatic pregnancies was twice as high as in the reference population. They hypothesized that bile acids can produce surfactant depletion in the alveoli [26] so the risks and benefits of preterm delivery must be considered individually. Also Bacq et al. concluded in their meta-analysis that UDCA therapy might benefit fetal outcomes [16]. It reduces fetal distress and fewer neonates in intensive care unit.

Caesarean section rate in our hospital during the study period varied between 13.9 and 17.4 % and in the study cohort the rate was 15 %. Also the vacuum extraction rate was 5.8–7.3 % in the hospital and in this study 5.5 %. Most deliveries were induced and early term delivery did not increase the incidence of caesarean section or vacuum extraction. Chappell et al. 2012 also reported planned early termed delivery not to increase caesarean section rate significantly [8]. In a recent prospective study of Geenes [27] et al. demonstrated significant increased risks of adverse perinatal outcome in severe ICP, so close antenatal monitoring of pregnancies affected by severe ICP is supported.

There were no stillbirths in our study material. Also other recent investigations show a marked decrease in fetal complication rates probably due to a greater awareness of the disease, experienced management and treatment [4, 19]. In our department the dose of the UDCA is quite low, but the perinatal outcome was good and maternal side-effects were minimal. We still recommend close fetal status monitoring in all cases of ICP. According to our study low dose UDCA treatment and induced delivery are safe and recommended as a treatment with ICP. Patients without the medication need also close antenatal surveillance.

## Conclusion

Low dose UDCA treatment reduces maternal liver function tests, is well tolerated by pregnant women and no fetal or neonatal side effects could be detected. Majority of the labours were induced. ICP is associated with preterm birth.

## Abbreviations

UDCA: Ursodeoxycholic acid
ICP: Intrahepatic cholestasis of pregnancy
Ctg: Cardiotocography
ALT: alanine aminotransferase
BA: bile acid
